# Production of Cellulose Nano-Fibers and Its Application in Poly-Lactic-Acid: Property Improvement by New Types of Coupling Agents

**DOI:** 10.3390/polym14091887

**Published:** 2022-05-05

**Authors:** Maria Elena Lozano Fernandez, Norbert Miskolczi

**Affiliations:** Research Centre of Biochemical, Environmental and Chemical Engineering, MOL Department of Hydrocarbon & Coal Processing, Faculty of Engineering, University of Pannonia, Egyetem u. 10, H-8200 Veszprém, Hungary; lozano.elena@mk.uni-pannon.hu

**Keywords:** poly-lactic-acid, cellulose nano-fiber, coupling agents, mechanical properties, improvement

## Abstract

Poly-lactic-acid is a biopolymer that can be an attractive alternative to replace petroleum-based polymers. It has advanced mechanical properties, melts easily with less energy consumption, and can be used to produce biodegradable plastics using renewable sources. However, some of the properties of poly-lactic-acid are inferior to those of traditional polymers: e.g., intensive farming is necessary for high agricultural yield, the composting needs special conditions, it is difficult to blend with other commonly used plastics, expensive, high permeability, etc. Therefore, the present work seeks to improve the structure and mechanical properties of the poly-lactic-acid incorporated by cellulose nano-fibers obtained from rice straw by a chemical acidic treatment. The fibers were incorporated into the poly-lactic-acid polymer matrix in a concentration of 1% by two-roll mill. To improve the incorporation of the fibers in the matrix, different coupling agents were used: PE-g-MA, vinyl trimethoxy silane, polyethylene-glycol with different molecular weight, and two types of experimentally synthetized α-olefin-maleic anhydride-based copolymers. The properties of the final composite could be improved, however those depend on the coupling agent to be used. The improving effect of the tested chemicals had been depended on the temperature. Based on structure analysis, both chemical and physical interactions were proposed between the cellulose nanofiber and polymer matrix. The thermogravimetric and viscosity results well represented the softener effect of the used chemical agents.

## 1. Introduction

The petroleum-based polymer market is growing constantly, due to the large possibilities of application in the packaging, automotive, construction, and other sectors [1,2]. Due to their advantageous properties, biopolymers are widely used in the packaging industry, medical application, and many other engineering fields. Among these research and application areas the bio-nanocomposites have a great opportunity, which can also be used to promote the development in the circular economy. Bio-nanocomposites are the mixtures of bioplastics and nano-sized materials. The main opportunity of biopolymers are their favorable mechanical properties, the sustainability, or even the biocompatibility and biodegradability [1,2,3]. Furthermore, authorities are creating regulations and encouraging the industry to produce materials and follow procedure that support the protection of the environment, enhance sustainability, and decrease the large amounts of plastic waste. Poly-lactic-acid (PLA) is a polymer that could be a potential alternative for the substitution of petroleum-based polymers, which can be obtained from renewable resources. Depending on the polymer configuration, PLA can be biodegradable [1]. However, it presents some disadvantages to be compared with traditional polymers; e.g., it is a brittle material with less than 10% of elongation at break, their gas barrier is poor, and it is highly hydrophobic. Therefore, the process ability is more difficult and the product performance in some cases is lower, compared with petroleum-based polymers [2,3,4,5].

The properties of PLA should be improved by its modification or reinforcement by using different techniques, such as surface modification, using coupling agents, by plasticizers or blending other polymers [5,6,7,8]. One of the alternatives for improving the properties of the PLA is the addition of nano-cellulose as a reinforcement, which is an attractive material in comparison with others due to its high availability in nature and sustainability [3,9]. The nano-cellulose can contribute to improving the mechanical properties, behaving as a nucleating agent or as cross-linking component [10,11].

The cellulose is a highly crystalline material with a high surface area which can be easily modified chemically due to the -OH groups in the structure that can react forming different bonds [4,12,13,14]. The arrangement and the axial orientation of the fibers provide better tensile properties such as high modulus and strength [4,12]. It was found that the mechanical properties (such as tensile strength, modulus, and elongation) can be significantly improved by the addition of 1–5% nano-cellulose to the PLA [9].

The uniform and good dispersion of the nanomaterial in the polymer matrix, with strong interactions between the phases, are key factors. The best mechanical properties of the composites that should be obtained by particles are dispersed in the matrix, interacting with H bonds [10]. The compatibility of phases is basically affected by the solubility parameters (polarity of the components), and interactions between the interfaces (hydrophobic and hydrophilic character) [4,15,16,17].

The highly crystalline nano-cellulose can be obtained from natural sources, e.g., cotton, wood, wheat straw, hemp, rice straw, flax, etc. It has excellent mechanical properties (e.g., high tensile modulus and stiffness) and biocompatibility, high surface area (>100 m^2^/g), and good thermal stability. Due to the active -OH group, it can be modified chemically. The size and structure of the nano-cellulose mainly depend on the feedstock, and the processing parameters. Depending on the nano-cellulose structure, it should be nanocrystals (small, highly crystalline), nanofibrils (moderate crystallinity), or even bacteria cellulose (less crystallinity with random orientation of micro-fibrils). The nano-cellulose can be applied in many purposes, and the market is rapidly developing [18]. Regarding the nano-cellulose production, it can be obtained by chemical treatment, mechanical, chemo-mechanical, and biological methods [4,19]. The most popular method is the chemical treatment. In general, high temperature, long residence time, and high concentration of acid lead to shorter nano-cellulose [10,20].

The nano-cellulose presents high polar and hydrophilic character, while the PLA is apolar and hydrophobic [3,4,10]. Due to the difference in polarity and solubility, the formation of bonds and adequate dispersion can be limited and need specific conditions and techniques of processing [10]. The non-adequate dispersion can lead to agglomeration and a negative impact on the composite properties [9]. Better properties should result in an effective stress transfer between the different phases. The interfacial forces should be both physical and chemical, which refers to the strength of linkage between the fiber and matrix. It is known that strong interaction should be found in the case of positive and high mixing enthalpy, however, due a to entropy effect, the too-high mixing enthalpy can lead to phase separation. The positive effect of the compatibilizers is explained by the fact that they can affect both the microphase and macrophase, which resulted in significant decreasing of interfacial tension [21]. To improve the dispersion and solve the compatibility issues, some treatments or surface modification can be performed, including the use of coupling agents, the modification of nano-cellulose surface, physical adsorption of processing aids, such as surfactants, or chemical grafting [5,6,7,8,9]. The coupling agents are molecules with two functional groups, which have the ability to react with both nano-cellulose and the polymer matrix [8]. Regarding the chemical effect of the compatibilizers, the free functional groups are particularly important because, with appropriate chemical structures, they can form strong chemical bonds between the various components of the composite. The commonly used coupling agents are organosilanes, acid functionalized polymers or groups derived from maleic anhydride and acrylic acid, which can form strong covalent or hydrogen bonds [8,22,23,24,25].

It was also demonstrated that the fibers act as points of interconnection between cellulose and polymer during the incorporation of the cellulose to the PLA matrix. The proper dispersion of the nano-cellulose in the PLA matrix can reduce the amorphous content, increase the crystallinity, and as a result, better mechanical properties of the composite should be reached [26]. In addition, the viscoelastic character of the composite was also modified, because the cellulose added to the PLA matrix affected the viscous or elastic behavior of the material [27,28,29].

The aim of this work was the preparation of waste-based nano-cellulose (NC) and its application in PLA. Furthermore, to improve the interfacial forces between the nanofiber and PLA matrix, different types of coupling agents were tested (among them two new experimentally synthetized α-olefin-maleic anhydride-based copolymers). The properties of cellulose nanofiber/PLA composites (NC/PLA) were followed through their composition and mechanical properties. The experimentally synthetized additives have not previously been used in nano-cellulose/PLA systems. Furthermore, the effect of widely used compatibility methods (e.g., silane-based agents and PE-g-MA) have been also compared with that of experimentally synthetized compatibilizers.

## 2. Materials and Methods

### 2.1. Materials

For the nano-cellulose synthesis, biomass waste (rice straw) was used, which is rather lignocellulosic biomass, with a vast amount of crystalline cellulose. According to ultimate analysis, it contains 48.7% oxygen, 41.9% carbon, 8.7% hydrogen, and 0.7% nitrogen. The ash content was 15.5%, while the moisture content and fixed carbon content was 3.5% and 16.1%, respectively.

In this work, PLA was used as matrix material for nano-cellulose reinforced composites. The main properties of the used PLA are summarized in Table 1. The tensile and flexural properties of samples were investigated by an INSTRON 3345 universal tensile testing machine with 50 mm/min crosshead speed. A CEAST Resil Impactor was used to follow the resistance of the composites against dynamic load using unnotched specimens.

The neat PLA had 16.8 MPa tensile strength, 975 MPa tensile modulus, 6.2% tensile elongation, and 25.7 kJ/m^2^ Charpy impact strength at 20 °C. These properties were significantly affected by temperature. For example, at lower temperatures both the tensile and flexural strengths of neat PLA were higher, and the polymer tends to be more rigid. However, the elongation at break and Charpy impact strength decreased as function of decreasing temperature.

Different materials and methods have been used to improve the interfacial forces between the nano-cellulose and PLA matrix, and to achieve more favorable mechanical and other properties (Table 2). Commercial vinyl-trimetoxy-silane, commercial polyethylene grafted maleic-anhydride, two types of polyethylene-glycol, and two experimentally synthesized α-olefin-maleic anhydride copolymers were used to achieve better interfacial forces between the disperse and bulk phases. The background of the compatibilizing additive synthesis method is described in WO/2009/050526 document, and more details about the synthesis is available in earlier publications [7,22,23]. The OMAC was synthetized by the using of maleic anhydride, mixture of C_20_-C_26_ olefins and initiator, while the α-olefin-maleic anhydride copolymer further reacted with C_16_ alcohol during the production of OMACE.

### 2.2. Nanocellulose Production

A method based on earlier publications was used for nano-cellulose synthesis [17,27,30]. The rice straw was grinded in a laboratory-scale equipment (DIPRE GRS182/A-9) and dried in a laboratory drying chamber at 40 °C for half day. The crashed and dried rice straw was pre-treated in an alkali solution to remove lignin and hemicellulose from rice straw fibers. The rice straw was immersed in 4% NaOH solution (using 1:10 ratio of rice straw and alkali solution) at 45 °C for 120 min, under constant stirring. Then, the suspension was filtered, and the solid fraction was rinsed with water several times.

To reduce the fiber diameter, produce a homogeneous product, and improve their properties a bleaching process was performed. After the alkali treatment, the wet solid fraction was bleached with two times of equal parts of a solution of 4% NaOH and 7% H_2_O_2_ at 45 °C for 2 h. Then the solids were recovered by filtration, washed with water, and treated with 64% H_2_SO_4_ solution at 20 °C for 90 min using manual stirring. At the end of the reaction, the mixture was cooled down by iced water.

**Figure 1:**
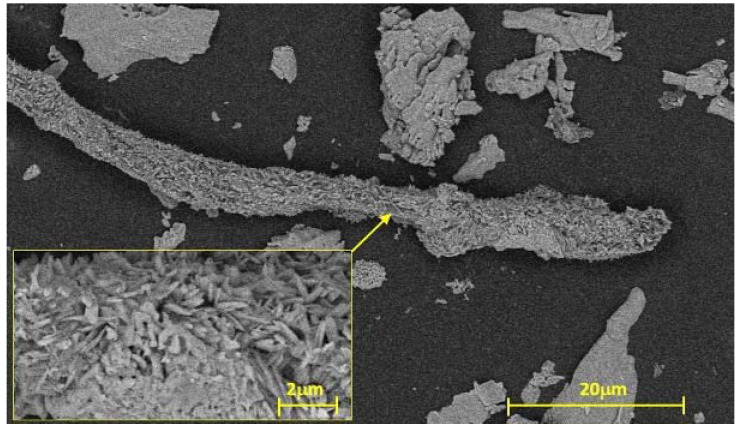
The SEM micrograph of the nano-cellulose.

The hydrolyzed product was separated by a centrifuge (B BRAUN BIOTECH SIGMA 2K15) at 10,000× *g* rpm for 10 min and washed with distilled water. Finally, the solid product was dried in an air-circulating chamber at 40 °C for 240 min. The structure of the obtained nano-cellulose was analyzed by SEM method (FEI Thermo Fisher Apreo S LoVac instrument, Brno, Czech Republic). In the micrograph of the treated material, it can be observed that the size of the elemental fibers was in the nanoscale (Figure 1).

The structure of the nano-cellulose contains long micro-rods and small packages, which most likely are formed by a network of macrofibrils and microfibrils. The nano-cellulose elementary fibers produced micro-cellulose fiber bundles. The size of the elementary nano-cellulose fibers is really small because the average diameter is around 10–100 nm, while the length is in the range of 100 nm to some μm. Similar structures were obtained by Nasri and Khenblouche, treating rice straw and retama rateam by a chemical method [31,32]. The structure presents some wrinkles that confirm the remotion of lignin, hemicellulose, and other impurities from the initial structure. A similar phenomenon was also demonstrated by Song et al., in which fibers from Calotropis fruit were obtained [33]. Smaller, pillow-like particles can be also observed, which are very similar to nano-cellulose crystals obtained from cotton by Khili [34]. The morphology of the structure suggests that the material is composed of crystalline and amorphous regions.

### 2.3. Manufacturing of Nano-Cellulose/PLA Composites

A laboratory two-roll mill (Lab Tech LRM-S-110/T3E, Labtech Ltd., Samut Prakan, Thailand) was used for mixing the nano-cellulose and PLA. The temperatures of the rolls were 180 °C and 130 °C. Then, 1 mm × 200 mm × 200 mm sheets were obtained by hot press mold at 180 °C using 1035 bar pressure. Finally, specimens with dimension of 1 mm × 10 mm × 100 mm were cut from composite sheets for further testing.

Table 3 summarizes the composition of NC/PLA composites. According to literature, in general 1–3% of nano-/micro-cellulose is added to the polymer matrix [4,11]. However, problems of agglomeration were also reported in cases of higher reinforcement content, therefore 1% of nano-cellulose was selected and used in this work.

### 2.4. The Structure of NC/PLA Composites by Fourier Transformed Infrared Spectroscopy and Differential Scanning Calorimetry Analysis

To investigate the chemical composition of specimens, TENSOR 27 type Fourier Transformed Infrared (FTIR-ATR) spectrometer equipped with an Attenuated Total Reflectance (ATR) accessory (Ge crystal) was used (number of 32 scans with resolution 3 cm^−1^). The FTIR spectra was recorded in the range of 400 and 4000 cm^−1^ at room temperature.

Differential Scanning Calorimetry (DSC) analysis was performed to study the behavior of the PLA composites using DSC 214 Polyma instrument. Samples were introduced in an aluminum perforated crucible and then were subjected to a four stage thermal cycle in which the following stages took place. The first heating was from 20 °C until 200 °C, after the samples were held at this temperature for 3 min to eliminate any residual crystallinity and erase the previous thermal history of the material. This was followed by a cooling stage until 20 °C, then the samples were heated for the second time. The heating and cooling rates used were 10 °C/min. From the curves, relevant information of the composites can be obtained, such as crystallization temperature (T_c_), glass transition temperature (T_g_), cooling crystallization temperature (T_cc_), melting temperature (T_m_), crystallization enthalp (ΔH_c_), cold crystallization temperature (ΔH_cc_), and melting enthalpy (ΔH_m_). The degree of crystallinity (X_c_) was determined by Equation (1).

(1) $$x_{c}=\frac{\Delta H_{m}-\Delta \mathrm{Hcc}}{\Delta H_{100}\left(\frac{\Phi \mathrm{PLA}}{100}\right)}$$

The ΔH_100_ is the enthalpy of melting 100% of crystalline polymer and the value for PLA corresponds to 93.7 J/g. The $\frac{\Phi \mathrm{PLA}}{100}$ is the weight percentage of the polymer matrix [35,36].

## 3. Results and Discussion

### 3.1. The Structure of NC/PLA Composites

#### 3.1.1. FTIR Analysis

**Figure 2:**
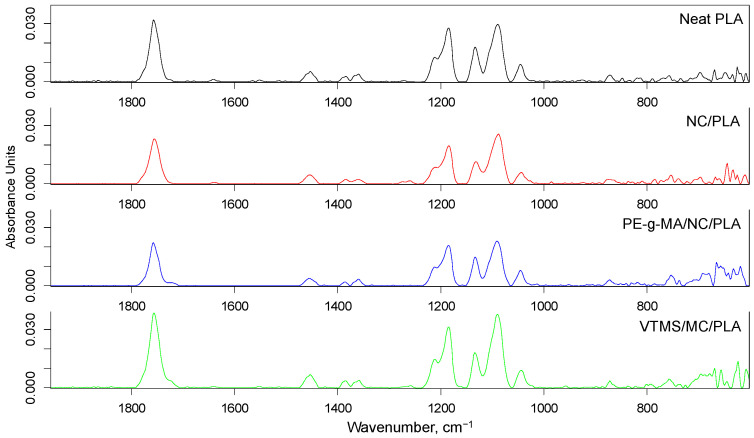
The FTIR spectra of specimens (neat PLA, NC/PLA, PE-g-MA/NC/PLA, VTMS/NC/PLA).

**Figure 3:**
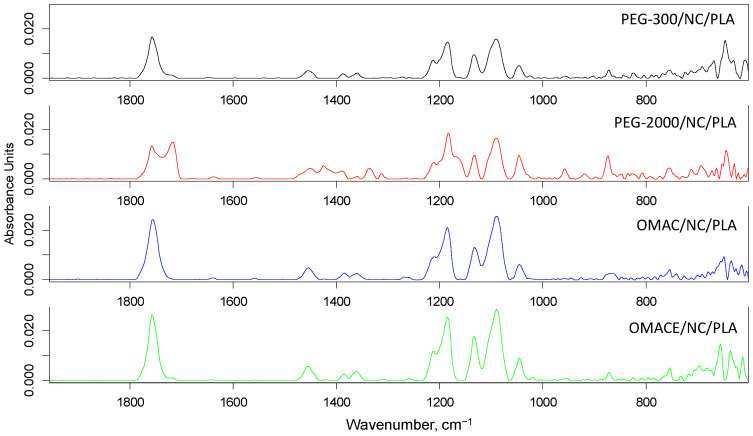
The FTIR spectra of specimens (PEG-300/NC/PLA, PEG-2000/NC/PLA, OMAC/NC/PLA, OMACE/NC/PLA).

The infrared spectra are shown in Figure 2 and Figure 3. The infrared bands between 1330 and 1460 cm^−1^ were associated to the -CH_2_- and -CH_3_ groups, while at 1730 cm^−1^ (C=O stretching vibration), at 1459 cm^−1^ (O-H stretching vibration), at 1145 cm^−1^ (C-O-C stretching vibration), and at 1014 cm^−1^ (C-O-H stretching vibration) to ester group. It is also known that C-O-C bonds in ester group show infrared activity at 940 cm^−1^ and 1225 cm^−1^, and the presence of cellulose should be also responsible for O-H, C-O, C-O-C, and C-O-H [36]. It was reported that bands at 1026 or 1056 and 896 cm^−1^ also refer to the presence of functional groups related to cellulose [36,37]. Due to presence of cellulose in the NC/PLA composite, a small additional peak (at 1024 cm^−1^) has appeared.

It was also concluded that the polyphenolic structure of lignin (conjugated aromatic rings and carbonyl groups) is responsible for different intensities in the range of 1500 to 1700 cm^−1^ [38,39,40]. Probably, small impurities from the rice straw remained in the structure. It can be concluded that the nano-cellulose is physically present in the matrix, but non-new chemical interactions are formed. In case of NC/PLA composite containing PE-g-MA, lower infrared activity was found between 1452 and 1360 cm^−1^, which was due to maleic anhydride chemical reaction with PLA. However, the -C=O carbonyl stretching (at 1722 cm^−1^) refers to the presence of anhydride groups. On the one hand, the aforementioned phenomena could be also found in the FTIR spectra of NC/PLA composites containing α-olefin-maleic anhydride copolymer and α-olefin-maleic anhydride ester copolymer. On the other hand, the anhydride group and carboxyl groups could also react with the -OH on the nano-cellulose fiber surface to obtain ester and hydrogen bond linkage.

Successful chemical reactions were proposed in VTMS/NC/PLA, because the Si-O-Si and C-Si-C groups are responsible for infrared bands between 800–950 cm^−1^ and at 663 cm^−1^, respectively. The application of PEG resulted in less chemical reactions than in the aforementioned cases, and there were no significant differences in infrared spectra of PEG-300/NC/PLA and PEG-2000/NC/PLA composites. It is an important observation that some new infrared bands were found both in the spectra of NC/PLA containing α-olefin-maleic anhydride copolymer and α-olefin-maleic anhydride ester copolymer (at 1132, 1045, 1089, 1211, 1184, 1360, 1313, 1427, 1452, 1750, and 1790 cm^−1^), which refers to the formation of new chemical bonds.

#### 3.1.2. Scanning Electron Microscopy (SEM) Analysis

**Figure 4:**
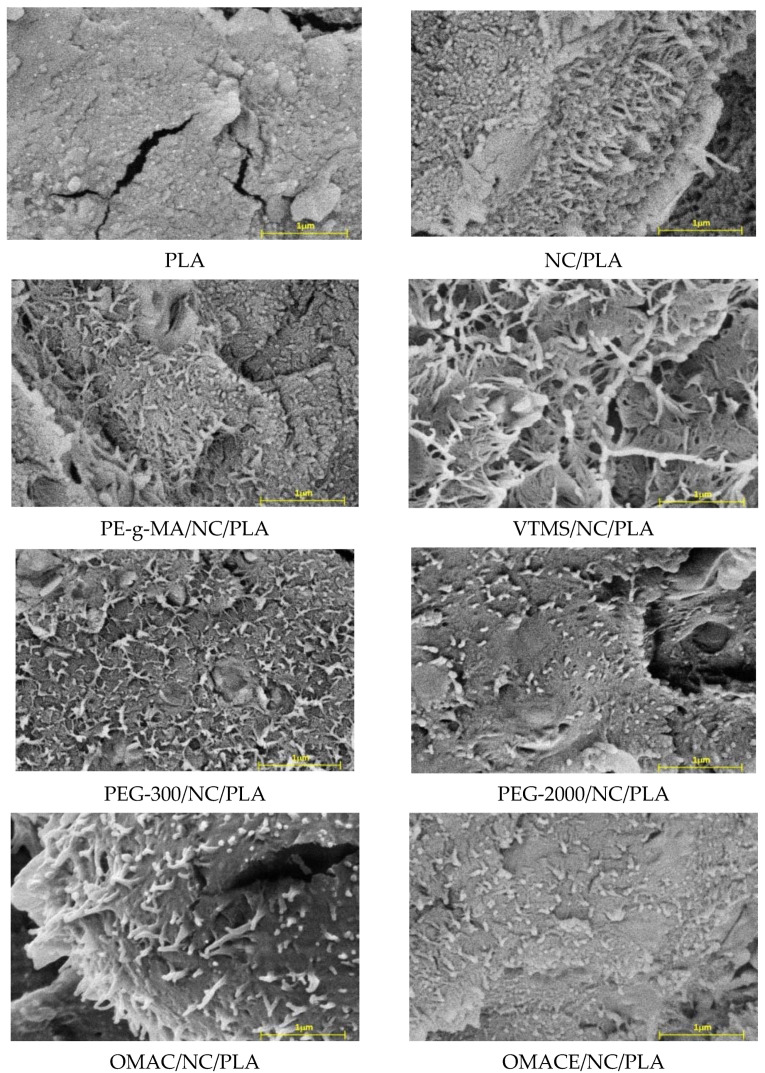
The SEM micrographs of samples.

The morphology of broken surface of different NC/PLA composites was investigated by a FEI Thermo Fisher Apreo S LoVac instrument. The SEM micrographs are summarized in Figure 4.

The SEM image of the fracture surface of the nano-cellulose-free neat PLA does not show the nano-cellulose fibers. The fracture surface in this case is smooth. However, the nano-cellulose fibers are well-visible, embedded in the PLA matrix in the case of SEM images of nano-cellulose-containing composites. The size of the nano-cellulose fibers is in the nanoscale range. The results also show that the nano-cellulose fibers assembled into microscale cellulose fiber bundles are well distributed in the PLA matrix, but no cellulose bundles should be found in the composites. This result refers to the processing parameters having fiber-dispersing property. It is worth mentioning that no significant differences (e.g., fiber elongation, fiber pull out, fiber agglomeration, or breakage) were observed based on SEM micrographs, and except NC/PLA, PEG-300/NC/PLA, and PEG-2000/NC/PLA compositions, the fibers are significantly more visible.

#### 3.1.3. DSC Analysis

**Figure 5:**
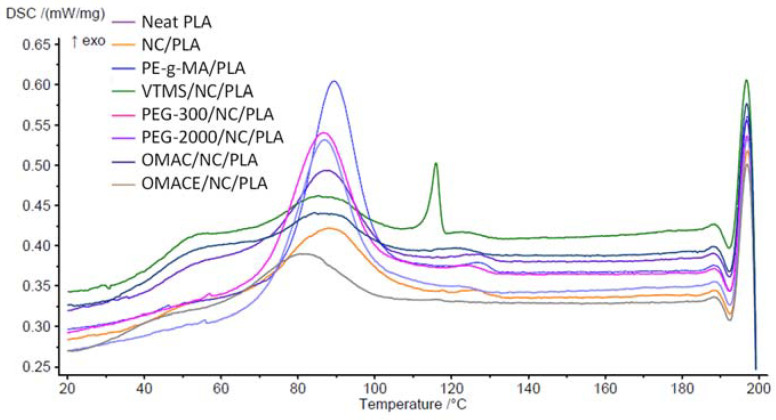
The DSC cooling curve.

**Figure 6:**
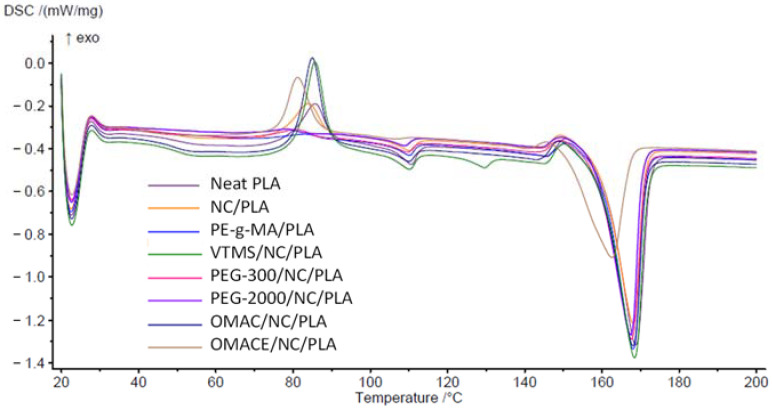
The second DSC heating curve.

The DSC cooling and second heating curves are shown in Figure 5 and Figure 6. The calculated properties are summarized in Table 4. It is well known that the low level of PLA crystallinity results in a limited thermostability [36].

From the cooling stage diagram (Figure 5), it was found that PLA has a crystallization temperature of 73 °C, while the T_c_ temperature of the new composites was lower except for VTMS/NC/PLA. The crystallization peak for the aforementioned composite appears very late (at 110 °C) in comparison with the neat PLA. For the rest of the samples, it was also observed that the ΔH_c_ was low, showing that this composite has a slow rate of crystallization. The PE-g-MA/NC/PLA, PEG-300/NC/PLA, and PEG-2000/NC/PLA show a significant increase in the enthalpy of crystallization with respect to neat PLA, which could be attributed to an enhancement of nucleation points and the mobility of the polymeric chains in the PLA. It was demonstrated in previous works that PEG groups exhibit a highly crystalline character by themselves, and when incorporated into the polymer matrix they can provide a synergic effect into the polymer matrix [35].

The T_g_ indicates the point where the polymer changes from a rigid state to a more flexible state, which can be obtained from Figure 6. The T_g_ was 51.8 °C in case of neat PLA. The T_g_ values of the composites change slightly with respect to PLA. Higher T_g_ corresponds to delaying in the mobility of the polymer chains, therefore the crystallinity of the material increases, and also the flexibility of the material is reduced [41,42]. It is expected that PEG-300/NC/PLA has higher crystallinity than PLA. In the opposite case, if the T_g_ is lower, the mobility increases, the VTMS/NC/PLA exhibits the lower value, suggesting that this is the most flexible material. The change in Tg and meting properties of nano-cellulose/PLA composites was also demonstrated by Kumar et al. [43].

The melting peak of the composites was approximately in the range of 161–168 °C. The Tm are not radically modified, they are slightly decreased, which indicates that the polymer undergoes a melting process at a lower temperature. The ΔH_m_ for all composites except VTMS/NC/PLA was reduced, which gives an idea that there is aggregation or interaction of a new element in the polymer matrix [44].

Additionally, a crystallization peak (exothermic) was formed in the thermograms between 78–85 °C. A factor that draws attention is that there is an absence of T_cc_ for PE-g-MA/NC/PLA. This can be interpreted and give elements to say that this composite behaves like a crystalline polymer [45]. With respect to ΔH_cc_, it is observed that PEG composites experience a decrease in their enthalpy. This can be due to a plasticization effect, which produces a fluidity to the material, and therefore a higher level of crystallization is produced. In the case of PE-g-MA/NC/PLA, no T_cc_ peak was found, and as a consequence, its level of crystallinity increases. The crystallinity (X_c_) of PE-g-MA/NC/PLA, PEG-300/NC/PLA, and PEG-2000/NC/PLA increased by 19.9, 12.3, and 14.6% with respect to PLA. A material with a higher degree of crystallinity indicates that the material is stiffer and stronger, but more brittle. The higher crystallinity was confirmed by Zhang et al., who demonstrated a significant increasing in “X_c_” value by adding of cellulose nano crystals to neat PLA [46]. The nano-cellulose should be a nucleating agent because it can promote the polymer chain mobility, which led to a higher degree of crystallinity [43,46,47].

#### 3.1.4. Thermogravimetric Analysis

**Figure 7:**
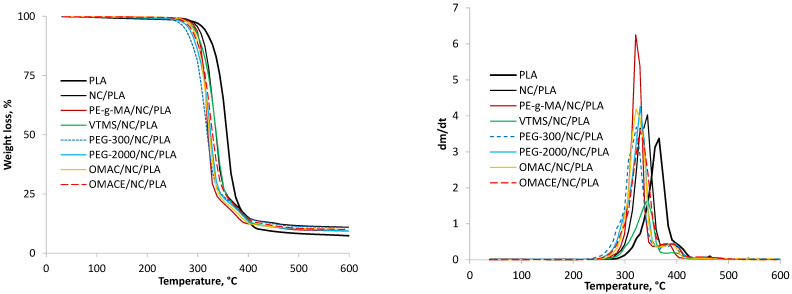
The weight loss and dt/dm curves of samples.

The thermogravimetric properties of nano-cellulose/PLA composites have been followed by a NETZSCH TG 209 F1 Libra type instrument using 30 °C/min heating rate in nitrogen atmosphere (20 mL/min). The weight loss of the samples and the dm/dt curves are summarized in Figure 7. The weight loss curve of neat PLA shows that it lost 2.2% of the initial weight up to 285 °C. The main weight loss step starts from 307 °C and finished up to 420 °C, where the weight loss was 90.1% of the initial weight. At 600 °C, the amount of residual material was 7.4%. Based on the dm/dt curve, the weight loss occurred at a maximum of 358 °C. The blending of nano-cellulose and various modifiers into the neat PLA can occur while visibly shifting, both in the weight loss and dm/dt curves, towards lower temperatures in each case. Regarding the blending of nano-cellulose into PLA, the main weight loss step of the composite began at 270 °C and finished up to 420 °C. The weight loss was <1% at 270 °C and 86.5% at 420 °C. Furthermore, the amount of residue was 10.9% at 600 °C.

It is worth mentioning that a well-visible shoulder on the dm/dt curve of neat PLA between 390 °C and 420 °C was found, which transformed into a well-separated peak for composites containing nano-cellulose and other chemical agents. Therefore, two peaks were observed on those dm/dt curves. In any case, the first peak appears at lower temperatures than in the case of neat PLA. For example, the first peak maximum was found at 343 °C for the NC/PLA sample, while the second peak was found at 397 °C. However, the final temperature of the weight loss step did not change significantly, as they remained in the temperature range of 415–420 °C for each composite. With the blending of various chemical agents into the NC/PLA composite, a significant reduction in the initial temperature of the weight loss step was concluded (into the range of 232–262 °C). On the one hand the lowest initial temperature was found in the case of PEG-300/NC/PLA. On the other hand, a shoulder appears below 300 °C on the dm/dt curve for this composite, indicating the presence of lighter molecules. A similar result was observed for PEG-2000/NC/PLA and OMACE/NC/PLA. As a result of the chemical modification, the peak values of the dm/dt curves also decreased, but in a different order than the decreases in the initial temperatures. It is also worth mentioning that, for example, there was only a small decrease in the location of the peak in case of VTMS/NC/PLA. It is important to mention that in general, the nano-cellulose has a positive effect on the thermal stability of the NC/PLA composite, therefore, the aforementioned deterioration in thermal stability was rather the consequence of the surface modifying agents [46].

### 3.2. Mechanical Properties

#### 3.2.1. Tensile Properties

**Figure 8:**
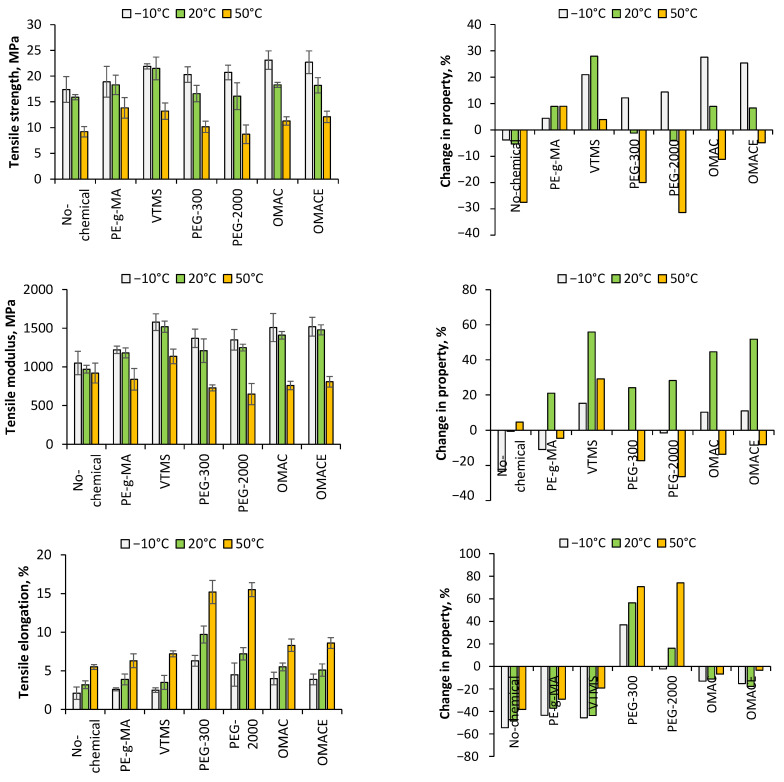
The tensile properties of nano-cellulose reinforced PLA composites and the property change.

The tensile properties of the composites are summarized in Figure 8. Due to weak compatibilization, lower values of both tensile strength and modulus of NC/PLA were found than the neat polymer (the neat PLA has 18.1, 16.8, and 12.7 MPa tensile strength at −10 °C, 20 °C, and 50 °C, respectively). The deterioration in tensile strength was higher, especially at higher temperatures. With increasing temperature, the tensile strength was 17.4, 15.9, and 9.2 MPa, which means deterioration of 3.9, 5.4, and 27.6%. The opposite tendency was observed with respect to the tensile modulus, e.g., deterioration of properties was observed at −10 °C (−23.4%), while an improvement of that was found at 50 °C (4.5%). In general, the nano-cellulose made the composite more brittle because the specific elongations were lower in all cases than those measured for neat PLA. However, the differences decreased with increasing temperature (from −54.3% to −38.2%). Chemicals added into the NC/PLA composites can improve the tensile strength at −10 °C in each case, while lower strength was found at 20 °C by the addition of PEG-300 and PEG-2000. Meanwhile, only the application of PE-g-MA and VTMS in NC/PLA composite was able to increase the tensile strength at 50 °C. The positive effect of grafted MA was confirmed by Ghasemi et al. [48]. Abdul Wahab et al. suggested that the grafted MA can create linkage between hydrophilic and hydrophilic constituents of the composites [49].

Regarding the tensile modulus, higher values were measured in all samples at 20 °C, while only the application of PEG-300 and PEG-2000 resulted in a lower modulus at −10 °C. Contrarily, only the VTMS/NC/PLA composite had a higher tensile modulus at 50 °C than that of neat PLA. VTMS/NC/PLA composite has the highest tensile strength and modulus measured at 20 °C (21.5 MPa and 1520 MPa). A similar result was concluded at 50 °C with tensile strength of 21.5 MPa and tensile modulus of 1580 MPa. Regarding VTMS, the improved tensile strength can be attributed to the siloxane groups. It was confirmed by earlier work that the siloxane group can create chemical linkage (e.g., cross linking) between the nano-cellulose fibers and the PLA polymer [37]. Rahmat et al. demonstrated that due to the partial cross linking of PLA, the tensile strength can be improved by VTMS in the case of nano-cellulose PLA composites [50]. Better mechanical properties of PLA containing agave bagasse derived cellulose nanoparticles, the surface of which was modified by 3-aminopropyl triethoxysilane silane, concluded in the experimental work of Robles et al. [51]. PE-g-MA has a ring with polar groups and a long chain with apolar property. The anhydride ring can increase the adhesion between the surfaces and form covalent bonds with the hydroxyl groups of nano-cellulose fiber. The long apolar chain could be linked to PLA.

It is important to remark that the application of experimentally synthetized α-olefin-maleic anhydride copolymer and α-olefin-maleic anhydride ester copolymer had a beneficial effect on the tensile strength and modulus at −10 °C. In case of both tensile strength and modulus of specimens measured at 20 °C, it can be concluded that decreasing tendency was found in property enhancement in the order of VTMS/NC/PLA, OMAC/NC/PLA and OMACE/NC/PLA composites. At 50 °C, deterioration in tensile modulus was measured when PEG-300 and PEG-2000 chemicals were added into NC/PLA composites. The tensile strength measured at −10 °C were 23.1, 22.7, and 21.9 MPa in case of OMAC/NC/PLA, OMACE/NC/PLA, and VTMS/NC/PLA composites, respectively, and 1510, 1520, and 1580 MPa tensile modulus was measured following the same order. Regarding the OMAC and OMACE, the anhydride ring and -OH groups are responsible for linkage to nano-cellulose fiber, while the alkyl chains should create linkage to PLA matrix.

The plasticizing effect of PEG is well known. Regarding the elongation, the difference between the NC/PLA and compatibilized NC/PLA can increased as a function of temperature. Apart from PEG-300/NC/PLA and PEG-2000/NC/PLA, the compatibilized NC/PLA composites had lower elongation values than that of neat PLA. However independently from the temperatures, higher values of elongations could be measured in each compatibilized NC/PLA composite, such as NC/PLA. The reason for this is also to be found in the molecular weight of the materials used, as they consist of smaller molecules compared to PLA. In addition, it is likely that the softening effect was more pronounced using PEG-300 and PEG-2000, because on the basis of tensile strength, it is well shown that weak interfacial forces were formed between the nano-cellulose and PLA matrix when PEG-300 and PEG-2000 were blended into the composite. This phenomenon was confirmed by Sompol et al. in the case of nano-cellulose/PLA composite and obtained by solvent-casting method in the presence of PEG [37]. The composite become more brittle, flexible, and ductile by the use of PEG.

#### 3.2.2. Flexural Properties

**Figure 9:**
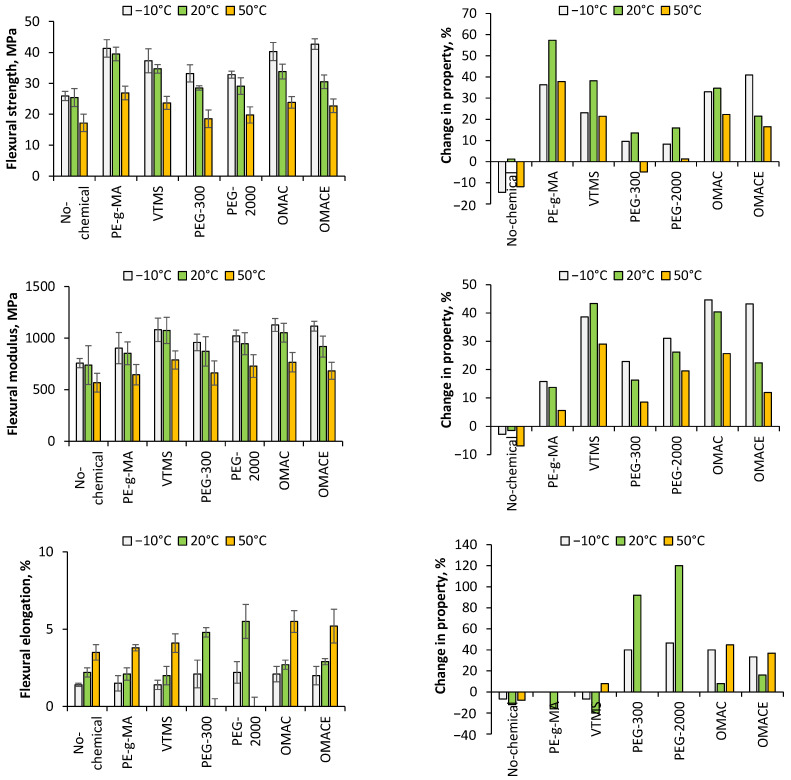
The flexural properties of nano-cellulose reinforced PLA composites and the property change.

The flexural properties of the composites are summarized in Figure 9. It is well shown that nano-cellulose/PLA without any other chemicals or linking agent had the lowest flexural strength, which was less than that of the neat PLA at −10 °C, and 50 °C (25.9 and 17.2 MPa, respectively). The decrease in these properties was 14.5% and 11.8%, related to the flexural strength of neat PLA. In case of sample at 20 °C, the strength increased slightly (1.2%), 25.4 MPa being the reported value. These measurements support that there is no good compatibility between the nano-cellulose fiber and PLA matrix without the presence of chemicals that serve as a bonding agent. The flexural strength of the rest of the composites can increase by the use of different modifying agents, except for PEG 300 at 50 °C, in which there is a decrease with respect to the value of neat PLA (−4.8%). The enhancement of the flexural strength should be associated with the number of loaded fibers in the matrix and with a favourable orientation and direction within the structure [39]. PE-g-MA achieved the highest flexural strengths of 41.3, 39.1, and 26.9 MPa at 10 °C, 20 °C, and 50 °C, respectively. The PEG modified nano-cellulose/PLA composites represented the less positive change comparing to the untreated NC/PLA. Regarding the OMAC/NC/PLA, OMACE/NC/PLA, and VTMS/NC/PLA composites, the two experimentally synthetized low molecular weight polymers showed significantly better flexural strength at −10 °C than the VTMS treated NC/LA. It is also an important observation that the upgrade in the flexural strength was more significant at 20 °C in all composites than for the rest of the temperatures.

Regarding the flexural modulus, it is clear that only the untreated NC/PLA composites had worse properties than the neat PLA (−2.8% (at −10 °C), −1.5% (at 20°), and −6.9% (at 50 °C)). The rest of the samples showed an improvement, which could be associated with the formation of networks between the phases [32]. The maximum values obtained from flexural modulus were 1117 MPa at −10 °C (OMAC/NC/PLA), 1075 MPa at 20 °C (VTMS/NC/PLA), and 787 MPa at 50 °C (VTMS/NC/PLA). It is important to note that the change of flexural modulus decreased as a function of temperature, which is an opposite trend to the optimum change of both tensile modulus and flexural strength. Meanwhile, there is less difference among the different specimens in flexural modulus than of that in flexural strength. In general, the VTMS and OMAC treated NC/PLA composite resulted in the best properties. Regarding the experimentally synthetized α-olefin-maleic anhydride copolymer and α-olefin-maleic anhydride ester copolymer, it is clear that the α-olefin-maleic anhydride copolymer looks better than the other. At 20 °C, more significant differences can be observed. This result corresponds to the stronger linkage between nano-cellulose and PLA matrix against flexural loading. Although PE-g-MA showed very favourable properties in improving the flexural strength of NC/PLA composites, it gave very poor results in terms of flexural modulus. The slight increase in tensile modulus, flexural strength, and flexural modulus as a function of cellulose nano crystals content in the PLA matrix was also demonstrated earlier [46].

Regarding NC/PLA, PE-g-MA/NC/PLA, and VTMS/NC/PLA composites, lower flexural elongation was found than in the case of neat PLA. Those results are similar to those presented in the case of tensile elongation. However, OMAC/NC/PLA and CMACE/NC/PLA showed positive changes in flexural elongation and negative in tensile elongation. It is also should be noted that the change in tensile elongation increased, while that of flexural elongation followed an optimum curve as a function of temperature. The plasticizing effect of chemical agents can be well monitored in NC/PLA composites containing polyethylene glycol.

#### 3.2.3. Charpy Impact Strength

**Figure 10:**
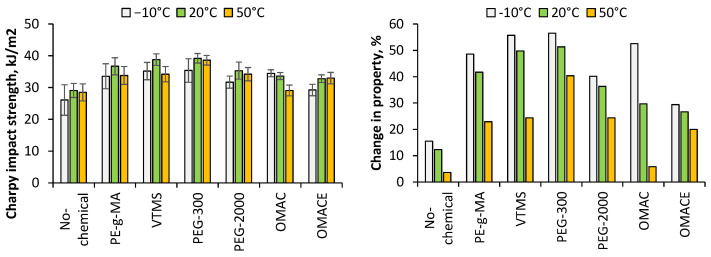
The Charpy impact strength of nano-cellulose reinforced PLA composites and the property change.

The Charpy impact strength of the composites is summarized in Figure 10. The measurement of Charpy impact strength is associated with the level of hardness of the polymer and the resistance against dynamic load. The results obtained from this test show advanced property in all cases, even in the NC/PLA that did not use any compatibilizer agent. The higher impact resistance values compared to PLA could be explained due to the addition of interfaces, and the fact that fibers can transfer the stress in a better way. Therefore, the composites present greater ductility. The Charpy impact strength changed as an optimum curve with maximum at 20 °C, however, the difference among the properties at different temperatures was in the narrow range. Contrarily, the change in impact strength related to the neat PLA followed a decreasing tendency as a function of temperature.

The best result was found in case of NC/PLA containing VTMS and PEG-300 at −10 °C and 20 °C, while PEG-300 was at 50 °C. Regarding the experimental additives, α-olefin-maleic anhydride copolymer showed a comparably positive effect compared to, e.g., the VTMS. Comparing the two polyethylene-glycols, it is clear that shorter polymer chain was favourable regarding the impact properties.

#### 3.2.4. The Dynamic Mechanical Testing

**Figure 11:**
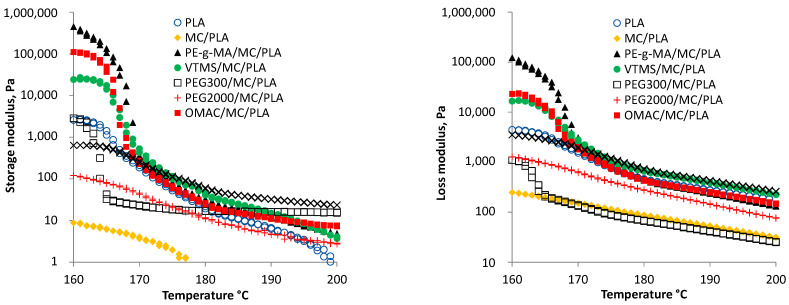
The storage modulus and loss modulus of composite samples.

The dynamic rheological properties of the specimens followed an oscillatory shear experiment using a parallel-plate rheometer (Anton Paar MCR). The linear viscoelastic region was determined using a dynamic strain amplitude sweep test from 0.001 to 100% at a frequency of 10.0 1/s, then the material responses of the samples were obtained by dynamic temperature ramp under a temperature range of 160 °C to 180 °C. Figure 11 depicts the storage modulus (corresponds to elastic response) and loss modulus (corresponds to viscous part) of composites. Results show that the blending of nano-cellulose into PLA matrix significantly affects the viscoelastic property, however the storage modulus could be enhanced by compatibilizers. This result refers to strong interactions among the constituents of composites, e.g., through the interaction of hydrogen and hydroxyl groups. Similar results were demonstrated by others using different chemical agents [45,52].

The storage modulus significantly and suddenly dropped between 160 and 170 °C. Following that, the storage modulus changed significantly less between 170 and 200 °C as a function of temperature. In general, higher mobility of polymer chain resulted in a rapid decrease in storage modulus. The PE-g-MA/NC/PLA, OMAC/NC/PLA, and VTMS/NC/PLA had higher storage modulus than the neat PLA, while the other had less. The softening behavior of polyethylene-glycol is well represented through the storage modulus because there is a rapid decreasing between 160 and 165 °C, however, after the rapid decrease, the rate of change slows down significantly and after 190 °C, the PEG-300/NC/PLA composite has the second highest storage modulus value. At higher temperature (>195 °C), the order of storage modulus is significantly different than that at lower temperature, because from 195 °C the following order was found: OMACE/NC/PLA, PEG-300/NC/PLA, OMAC/NC/PLA, PE-g-MA/NC/PLA, VTMS/NC/PLA, PEG-2000/NC/PLA, neat PLA, NC/PLA. Regarding the loss modulus, the PE-g-MA has the highest value at a low temperature region and the aforementioned significant drop was found between 160 and 170 °C, however, the loss modulus decreased very suddenly in the case of PEG-300/NC/PLA, but this was contrary to storage modulus. In that case, the order of composites at higher temperature was the following: OMACE/NC/PLA, VTMS/NC/LA, neat PLA, OMAC/NC/PLA, PE-g-MA/NC/PLA, PEG-2000/NC/PLA, NC/PLA, and PEG-300/NC/PLA.

## 4. Conclusions

In this work, the production of waste-based nano-cellulose and its application in PLA composite was investigated. To achieve better interfacial forces between the nano-cellulose fiber and PLA matrix, different chemicals and two new, experimentally synthetized α-olefin-maleic anhydride-based copolymers were tested. The experimental results showed that nanoscale elementary cellulose fibers could be produced by the acid treatment of rice straw biomass. The nanoscale elementary fibers were clustered into larger microfiber bundles. Based on FTIR, DSC, and TG analyses, clear differences were found among the composites prepared in different ways. The various compounds used to improve the fiber compatibility in each case reduced the flow properties of PLA. PEG, regardless of its chain length, significantly increased the crystallinity of the composite. However, the elasticity of these composites increased the most significantly. Both physical and chemical interactions between nano-cellulose and PLA were proposed. Based on the mechanical tests, the organosilane compound and the experimentally synthetized α-olefin-maleic anhydride-based copolymers showed favorable results, however, the effect was highly dependent on the temperature of the test. In the case of experimental α-olefin-maleic anhydride-based copolymers, the free carboxyl group was advantageous in the forming of chemical bonds with cellulose molecules.

## Figures and Tables

**Table 1:** The main properties of the used PLA.

Property	Standard	−10 °C	20 °C	50 °C
**Tensile strength, MPa**	MSZ EN ISO 527-2:2012	18.1	16.8	12.7
**E-modulus, MPa**	MSZ EN ISO 527-2:2012	1370	975	880
**Elongation at break, %**	MSZ EN ISO 527-2:2012	4.6	6.2	8.9
**Flexural strength, MPa**	MSZ EN ISO 14125:1999	30.3	25.1	19.5
**E-modulus, MPa**	MSZ EN ISO 14125:1999	780	750	610
**Elongation, %**	MSZ EN ISO 14125:1999	1.5	2.5	3.8
**Charpy impact strength, kJ/m^2^**	MSZ EN ISO 179-2:2000	22.6	25.9	27.5

**Table 2:** The used chemicals for coupling of NC/PLA composites.

Symbol	Name	Source	Chemical Formula
**PE-g-MA**	Polyethylene grafted maleic-anhydride	n.a.	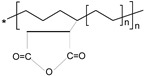
**VTMS**	Vinyl-trimetoxy-silane	Sigma-Aldrich	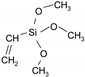
**PEG-300**	Poly (ethylene glycol), average molecular weight 300	Sigma-Aldrich	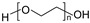
**PEG-2000**	Poly (ethylene glycol), average molecular weight 2000	Sigma-Aldrich	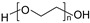
**OMAC**	α-olefin-maleic anhydride copolymer	Synthetized at the University of Pannonia	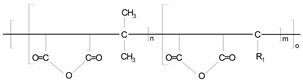
**OMACE**	α-olefin-maleic anhydride ester copolymer	Synthetized at the University of Pannonia	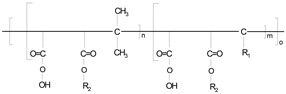

**Table 3:** The composition of NC/PLA composites, in (*w*/*w*)%.

Constituent	No-Chemical	PE-g-MA	VTMS	PEG-300	PEG-2000	OMAC	OMACE
**PLA**	99	98	98	98	98	98	98
**Nano-cellulose**	1	1	1	1	1	1	1
**PE-g-MA**	-	1	-	-	-	-	-
**VTMS**	-	-	1	-	-	-	-
**PEG-300**	-	-	-	1		-	-
**PEG-2000**	-	-	-	-	1	-	-
**OMAC**	-	-	-	-	-	1	-
**OMACE**	-	-	-	-	-	-	1

**Table 4:** The main properties of composites based on DSC result.

	T_c_	ΔH_c_	T_g_	T_cc_	ΔH^cc^	T_m1_	T_m2_	ΔH_m_	X_c_
**PLA**	73.0	9.2	51.8	85.6	8.5	161.6	168.7	43.7	37.6
**PLA-NC**	66.9	10.0	-	83.8	5.5	160.1	168.0	37.6	34.6
**PE-g-MA/NC/PLA**	69.8	21.8	51.8	-	-	159.7	166.7	41.4	45.1
**VTMS/NC/PLA**	110.4	1.4	41.8	85.3	11.1	159.8	167.2	44.4	36.3
**PEG-300/NC/PLA**	61.2	21.5	57.3	80.2	1.3	158.3	166.3	40.1	42.3
**PEG-2000/NC/PLA**	67.9	18.1	50.1	78.4	0.5	158.9	167.4	40.1	43.1
**OMAC/NC/PLA**	60.0	5.3	45.3	85.0	12.2	159.9	167.9	42.7	33.2
**OMACE/NC/PLA**	60.5	7.6	-	80.6	9.1	150.6	161.6	31.1	24.0

## Data Availability

Not applicable.
